# A Treatise on Insanity, and Other Disorders of the Mind

**Published:** 1835-07-01

**Authors:** 


### A TREATISE ON INSANITY, AND OTHER DISORDERS AFFECTING THE MIND. By *J. C. Prichard*, M. D.

[Article the Second, continued from p. 448 of No. 4.]

OUR notice of Dr. Prichard's work, in our last number, stopped short at the end of the third chapter, and at a natural division of the subject. The fourth chapter is on the CAUSES of INSANITY. These he divides into two classes—the predisposing, or antecedent, and the structural changes observed on dissection. It will strike the pathologist at once, that rational doubts may be entertained respecting the propriety of placing these post mortem phenomena revealed by the scalpel, among the *causes* of insanity—or of any disease. Thus, on opening a body after death by pleurisy, the sero-purulent effusion, and even the thickening of the pleura itself, could hardly be considered the cause of the pleurisy. The same reasoning might apply to an infinity of diseases. On the other hand, we are sometimes obliged to content ourselves with the appearances after death, not being able to ascertain the antecedent causes or links of the morbid chain. Fever may be adduced as an example—and perhaps the disease now under consideration—INSANITY. Our author adopts the division of causes into moral and physical, prefacing his subject with some remarks on predisposition. This is branched into constitutional predisposition, whether hereditary or self-acquired—influence of sex, age, climate, &c.—thirdly, external peculiarities of organization, form, complexion, &c.—fourthly, accidental diseases, as of the brain—and lastly, the influence of education, moral and physical, in modifying the "organization of the body and predispositions of the mind." On all these points our author collects the usual and necessary information befitting an elementary treatise. On few of these points shall we touch. Dr. P. conceives that a predisposition to insanity must exist in the individual: otherwise that the exciting or occasional causes would call forth some other disease rather than alienation of mind. Intemperance is adduced in illustration. Of the thousands that daily give way to this vice, only a very few become insane—apoplexy, paralysis, dropsy, and other corporeal maladies are much more frequently the result of inebriation. This predisposition may be transmitted from parent to progeny; or it may originate, *de novo*, in the individual. Dr. P. thinks it is of little consequence to inquire which of these two is the case. We apprehend that hereditary predisposition is more formidable than the recently-acquired. The intermarriages among relations have been accused, and perhaps justly so, as predisposing to mental as well as corporeal weakness. M. Esquirol has seen instances where frights, during pregnancy, have produced insanity in the child. It was observed, so far back as the time of Cælius Aurelianus, that the male sex were more frequently affected with insanity than the female. This does not accord with modern experience. In all France, the proportion of female to male cases of insanity is 14 to 11. In Italy the balance is on the other side. In Holland and Belgium the female lunatics predominate over the male, in the proportion of 34 to 29. In Great Britain and Ireland the proportion is 13 males to 12 females. It is difficult to account for these differences in the proportions of the sexes. In America there are nearly two men insane for one woman. Probably the addiction of the male sex there to ardent spirits, may help to account for this.

"On summing up the results of his inquiries, M. Esquirol has shown that in a sum total of 76,526 lunatics, confined, though not all at the same period, in asylums or hospitals in various parts of the civilized world, there were 37,825 males and 38,701 females. Thus the proportion of males to that of females is, a fraction being neglected, 37 to 38. This difference is so much the less considerable, as in the general population the number of males somewhat exceeds that of females. Yet, small as it is, it is sufficient, as M. Esquirol observes, to refute the assertion of Cœlius Aurelianus, who supposed that women are less subject to insanity than men." 165.

Insanity is rarely seen before the age of puberty ; but after that period, it may be seen in all the phases of life.

" In the vigour of youth," says M. Esquirol, " mania breaks out with all its varieties of excitement; melancholia is more frequently the lot of the middle period of life, and dementia chiefly threatens advanced age." 166.

According to M. Georget, insanity is most frequent between the years 30 and 40 of life—next in frequency is the period between 20 and 30—and thirdly, between 40 and 60. There is some inconsistency in this statement evidently.

The melancholic temperament is proverbially prone to insanity, and one attack proves a predisposing cause of subsequent ones. The author gives us a short article on education, as a predisposing cause of mental derangement, from which we shall quote one or two short sentences.

" There are two different points of view under which the injurious effects of wrong education may be considered. By too great indulgence and a want of moral discipline, the passions acquire greater power, and a character is formed subject to caprice and to violent emotions : a predisposition to insanity is thus laid in the temper and moral affections of the individual. The exciting causes of madness have greater influence on persons of such habits than on those whose feelings are regulated. An overstrained and premature exercise of the intellectual powers is likewise a fault of education which predisposes to insanity, as it does also to other diseases of the brain."

" The second remark, on the regulation of mental exercise in young persons whose nervous systems are feebly constituted, has a more extensive bearing than on the subject of insanity. It brings forward a suggestion which is of very general interest in these times, in which mental exertion is stimulated to the utmost; and too great sacrifices are often made to the cultivation of intellect, or even to the mere acquisition of knowledge, while the education of the moral affections is considered as a matter of secondary importance." 173.

Dr. Prichard proceeds to the productive causes of insanity—moral and physical. In the former class are included all those circumstances " which exert an influence immediately on the mind." Physical causes are those which act immediately on the body, and secondarily on the mind, through the organic structures. Most writers, and our author among the rest, give the preponderance to *moral* causes—and the same might be urged in respect to more diseases than insanity. The influence of the mind on the body is as great as that of the body on the mind. Insanity is rare among savage tribes—it increases with civilization.

" The restraints imposed by social order, the diversity of interests which are excited in civilized communities, the mixed and diversified feelings which are called forth by a variety of sometimes arduous pursuits, long-continued griefs and anxieties, disappointment of hopes long cherished, causes which act powerfully on the moral affections rather than on the animal passions, particularly great and long-continued exertion of the intellectual powers ;—these are some of the most obvious traits which distinguish human life in the civilized state from the manner of existence peculiar to savage men. It is among these circumstances, as some celebrated writers have thought, that we are to look for the causes which are most influential in the development of mental diseases." 175.

Our author, however, is disposed to doubt the truth of these arguments, or, at least, to dispute the extent to which they ought to be carried. "I am not without suspicion," says he, "that there is something in the state of civilization which tends to promote the existence of that congenital state of bodily structure on which predisposition to mental diseases depends." This, after all, is merely questioning the *quo modo*, and leaving the *quo* where it stood. Of the moral causes of insanity, care and anxiety, grief and agitations of mind, are the most operative. M. Esquirol informs us that, "at the destruction of the old monarchy, many persons became mad through fright and loss of property. When the Pope came to France, religious maniacs were very numerous. When Bonaparte made kings, there were many kings and queens in the madhouses. At the time of the invasion of France by foreign troops, terror threw many into derangement. The Germans had experienced the same effects, at the æra of our irruptions into their country." The strong passions of the mind come next in order of potency. The former class are generally slow in their operation:—the passions, like whirlwinds, often do their work more quickly. Terror and violent rage are the most rapid of all other causes in dethroning reason. Celibacy must be considered as strongly predisposing to, if not causing insanity. In the Bicetre, in January 1822, out of 1726 females, 980 were unmarried—397 married—232 widows—5 divorced—53 not noted. These were all females. The males only amounted to 764, and were nearly in the same proportion as to the married and unmarried.

The fear of punishment, in a future state of existence, is a well-known cause—or, at all events, a well known phenomenon in insanity. We agree with Dr. P. in the following sentiments.

"As a matter of fact, there is reason to believe that the number of persons who become insane through the influence of religious hopes and fears, is much less considerable than it is generally supposed to be. The circumstance that the mind of a lunatic is occupied during the period of his disease with ideas and feelings connected with an invisible world, is no proof whatever that the derangement of his understanding was produced in the first instance by impressions related to the same subject. To a mind already prepared by disease to indulge fearful thoughts and gloomy forebodings, the unknown future opens a wide field which the imagination is likely to select, and it often dwells upon the evils which it anticipates in another stage of existence, when the original cause of derangement has been some misfortune of the present life, or perhaps some merely physical influence. It is the opinion of a writer, whose judgement on subjects of this nature deserves the highest regard, on account of the extensive research and the deep reflection with which he has investigated the history of mental disorders, that instances of the last-mentioned kind are in fact incomparably more numerous than those in which religious terrors have been the originating cause." 188.

The writer alluded to is Dr. M. Jacobs. There can be little doubt, however, that ranting and fanatical preachers have often unhinged the minds of weak and ignorant auditors—especially females, whose imaginations are more active, and nerves more sensitive than in the rough sex.

"In the kingdom of Naples," says M. Berthollet, "a custom exists of preaching in favour of missions by a particular set of priests. In order to animate the faith of believers, they accompany their orations with particular acts, which are often of such a nature as to produce too powerful an effect on weak minds.

They hold their hands over flaming torches, and whip themselves with scourges garnished with iron points. Their sermons are prolonged till the close of day, and the feeble glare of a few flambeaus heightens the effect of the scene. "One of these sermons gave occasion to the case I am about to describe. The subject was *hell:* to heighten the colouring of the frightful picture which the preacher had traced, he took a skull in his hand, and having raised a question as to the abode of the soul to which it belonged, he exclaimed, invoking it, 'If thou art in heaven, intercede for us ; if thou art in hell, utter curses.' He then cast it from him with violence." The lady, whose case is subsequently described in M. Bertholle's memoir, was instantly affected by a morbid change in the nervous system." 189.

Our author takes great pains to ascertain the comparative prevalence of insanity among different religions, or rather sects. The result is not very satisfactory—but one thing appears clear,—that religious insanity is less frequent among quakers than among any class of people.

Dr. P. next comes to the PHYSICAL CAUSES of insanity, which he treats of under the following heads, viz. Injuries of the Head—Insolation and Exposure to Heat—Metastasis—Inebriation—Sensuality—Intestinal Irritation—Uterine Irritation. These seven heads occupy only as many pages of the work, and contain nothing that would not suggest itself to the mind of any ordinary practitioner on glancing over the catalogue.

The fifth and sixth chapters, consisting of about 40 pages, treat of the morbid anatomy of insanity. Our author begins with the morbid appearances found in the head, and proceeds thence to those which are presented in other parts of the body. Morgagni, the father of pathological anatomy, relates but seven or eight cases of insanity, where dissection was performed. He found the cerebral hemispheres firmer, and the cerebellum softer than natural, in one or two instances. But Greding was the first who recorded an extensive series of observations on this point, being physician to a lunatic asylum in Germany, where great numbers of the insane are congregated. The following are the principal facts which he noted.

"1. In the *cranium.* 'Experience has proved,' as he says, 'that the skulls of almost all insane persons have a natural shape.' In 16 cases only of the whole number examined, viz. nearly 220, the forehead was contracted, the temples compressed, and the occiput large and expanded. In a few the head was elongated and compressed at the temples. Some had a head almost round, or of a square shape : these were epileptic idiots. Two had small heads, quite circular : these were epileptic madmen. Of 216 cases, including those of madmen, idiots, and epileptics, the skull was unusually thick in 167 : this fact was observed in 78 out of 100 cases of raving madness, and in 22 among 30 of idiotism. In many cases the cranium was remarkably thin. Holes were observed in the inner table in 115 out of 216 cases : in other instances bony projection from the inner surface.

2. *Membranes.*—Dura mater firmly adherent to the skull in 107 out of 216 cases ; in a few instances of a blueish black colour, thickened and partially ossified. Pia mater thickened and opaque more or less in 86 out of 100 cases of mania ; beset with small, spongy bodies in 92 out of 100 : these bodies were often united to the surface of the brain, and were in some instances the seats of ossific deposits.

3. *Brain.*—Cerebral substance softer than usual in 118 out of 216 cases : soft and pulpy in 51 cases of mania out of 100, as likewise in 19 out of 24 cases of melancholia, in 8 out of 20 epileptics, and in 16 out of 30 idiots. Those maniacs who had the cerebrum softened had the cerebellum still more soft and pulpy.

4. *Effusions.*—Between the dura and pia mater in 120 out of 216 cases; in 58 out 100 maniacs. Between the pia mater and the surface of the brain in 28 among 100 maniacs. Lateral ventricles in 29 very full of serum, in 23 ready to burst; in 10 among 24 melancholics astonishingly distended. Third ventricle quite full in 57 of 100 maniacs, and in 16 of 24 melancholics. Fourth ventricle ready to burst in 80 out of 100 maniacs, and quite empty in only 3: completely distended in every one of 24 melancholics.

*Other appearances.*—Plexus choroides in a nearly healthy state in only 16 out of 216 cases, thickened and full of hydatids in 96 out of 100 maniacs. Lateral ventricles either larger or smaller than natural in many cases. Softness of parts of the brain, as of the tubercula quadrigemina in some cases." 211.

Dr. Haslam has given us the result of 37 dissections, in not one of which were the brain or its membranes free from disease. M. Georget, whose researches are extensive and accurate, states the following as the most remarkable of the *post-mortem* appearances: viz. irregular conformations of cranium, the prominences on the right side being generally larger than on the left—general or partial thickening of the cranium—dura mater rarely changed—arachnoid often diseased, generally thickened—pia mater injected, or thickened and infiltrated with serum—volume of the brain sometimes less than the capacity of the skull—brain sometimes very hard—the white substance glutinous, elastic, and bearing distention—but most frequently the brain softened, the grey matter being pale and yellowish—convolutions separated by serosity—pia mater thickened—interior cavities of the brain sometimes much enlarged—in others, filled with a limpid fluid—cerebellum usually softer than the cerebrum—medulla oblongata and spinalis seldom altered in structure.

M. Pinel after observing that the dissection of lunatic heads displayed the same phenomena that are usually seen in other cerebral diseases, seemed to abandon the hope of discovering any pathological anatomy peculiar to insanity. Esquirol appears inclined to fall into the same opinion, though he carefully notes the morbid phenomena revealed by the scalpel. His conclusions are as follows:—

"The inspection of bodies of lunatics offers numerous varieties as to situation, number, and kind of morbid appearances. The lesions of the encephalon are neither in relation to the disorder of the mind nor to the maladies complicated with it. Some lunatics whose mental and bodily disease had given suspicion of extensive organic lesions, have presented but slight changes of structure in the brain, while others whose symptoms had been less severe have been the subjects of great and numerous alterations. But what disconcerts all our theories is that not unfrequently, even in the instance of patients who have passed through all the stages of insanity, and have lived many years under derangement, no organic changes whatever have been traced either in the brain or its containing membranes." 213.

He acknowledges, however, that in the examination of 199 bodies, he found 263 organic lesions of the brain and its meninges—46 of the lungs, the heart, or their coverings—and 113 lesions of the abdominal viscera. But to what, after all, does Esquirol's reasoning tend? Only to this—that disordered *function* does not always leave behind it an appreciable change of *structure*. In mental derangement, as in all other derangements, the organic change is the *effect*, and not the cause of the malady. The primary cause—the disorder—may go on for years, or for a whole life in some cases, without producing the changed structure revealed by post-mortem investigation. But there is every reason to believe, that the immediate seat of insanity is in the *brain* (or its membranes), the organ of the mind, although dissection discloses not the *effects* of the disorder. We know that the stomach may be long *disordered* before it becomes *diseased*—that functional disorder of the kidneys may exist for half, or the whole of life, without organic change being found after death. Why, then, should not the material organ of the mind experience the same fate? After all, M. Esquirol admits that the greater care now taken with dissections than formerly, discloses more morbid changes in the brain than his early dissections led him to expect.

"Hence (he says), few bodies of lunatics are now examined in which there are not proved to exist at the same time injections or adhesions of the meninges, softenings and tubercles in the brain, serous effusions in the cavities, &c. while at the period when we made our earliest investigations, we only kept account of obvious and manifest alterations." 214.

This speaks for itself. Bayle, who has still more recently prosecuted the investigation, comes to the conclusion, that the seat of insanity is not in the brain itself, but in the meninges—consisting of effusion, the result of inflammation, and, consequently, *compression of the brain*. It is hardly necessary to observe that, according to this view of the case, the actual cause is disturbance of the brain's function from compression. The inflammation and effusion are antecedent links in the chain of causation. The mental malady does not shew itself fully till the organ of the mind suffers. The investigations of M. Calmeil are peculiarly important. It is true that they were made in elucidation of the paralysis of insane persons; but this does not lessen their interest or value.

"1. Bones of the skull sometimes very full of blood, which fills the spongy tissue, reddening it, and exudes from the surfaces of the cranium when denuded and separated from the dura mater. This is mentioned merely as indicative of vascular turgescence often connected with it in the encephalon.

2. Vegetations or excrescences arising from the pia mater, with absorption of corresponding parts of the inner table of the cranium. These are granulations growing up from the surface of the pia mater, penetrating the dura mater, and causing absorption of the inner bony surface. As for the pathology of this appearance, it is remarked that infiltrations and thickenings of parts are found almost constantly under these excrescences of the pia mater.

3. Effusions of serosity in the great cavity of the arachnoid, in the ventricles of the brain, and in the cavity of the rachis. This is one of the most striking and uniform symptoms in persons who have died of general paralysis. The quantity of serosity varies. Six or eight ounces are often found in the cavity of the arachnoid. M. Calmeil attaches less importance to this phenomenon than many have done. The following are his reasons. 1. He has observed it to be wanting in some strongly marked instances of the disease. 2. He has frequently discovered similar effusions in the heads of individuals who had perished under dementia, without any symptom of paralysis even to the last. 3. In cases in which effusions were found of five or six ounces of serosity, the symptoms of general paralysis had been not less intense, or even more intense, than in others displaying effusions of twice that extent. 4. If the compression of the brain was so considerable as many have thought, the structure of its parts would display disorganization of some kind, but the structure of the convolutions, commissures, septum, &c. is uninjured. 5. In cases of chronic hydrocephalus of long duration the deposit of serosity has been enormous, without loss of locomotive power till the disorder reached its last degree. 6. If compression from such a cause acted mechanically, we should expect paralysis depending on it to affect all nerves equally or indifferently; no reason could be perceived why the motive faculty should be impaired first in the tongue, then in the muscles of the lower members, and lastly in the upper, as the fact is observed to be in general paralysis. 7. We certainly cannot imagine that, such a cause acting, the upper extremities would still retain their mobility unimpaired, after the total palsy of the lower limbs. For these and other reasons, M. Calmeil concludes that the symptoms of general paralysis are not, as M. Bayle supposed, dependent on compression of the brain, the result of effusion, but on the state of the encephalon which gives rise to such effusion, and chiefly to inflammation, of which the thickenings, adhesions, and vascular turgescence of the pia mater, and the peculiar condition of the cineritious substance otherwise afford sufficient proof.

4. Other morbid phenomena occurring in general paralysis with relation to the state of the membranes are, false membranes, sometimes organized, at others unorganized, between the laminæ of the arachnoid; encysted concretions with hemorrhages between the same laminæ: these phenomena are referred in like manner to inflammatory action, or to erethism in the capillaries of the meninges; simple hemorrhages in the great cavity of the arachnoid; serous infiltrations of the pia mater and the cerebral arachnoid, a phenomenon found in many diseases, but rarely to such a degree as in the paralysis of dementia; thickenings of the pia mater and the cerebral arachnoid; a high state of vascular injection of the same membranes.

5. Adhesions, either general or local, of the internal surface of the pia mater to the cineritious portion of the brain. This is not universally observed in cases of the disease described, although strongly marked. It is important, as giving an indubitable proof of inflammation affecting the surface of the brain.

6. A curious and no doubt important series of observations refer to the state of the grey and white substance.

The grey substance contiguous to the pia mater is softened, and has the consistence of the pulp of a rotten apple. This ramollissement extends to the depth of a quarter or half a line. This appearance is accounted for by the observations of M. Lallemand, who remarks that permanent accumulations of blood diminish the cohesion of any parenchyma; even muscular parts, spleen, lung, can easily be crushed when gorged with blood. In brains of persons destroyed by intense cerebral congestion, the corpus callosum, the septum lucidum, and other parts are found relaxed, and their consistence gives way to slight pressure; it is to accumulation of blood that the loss of consistence in the superficial grey substance in general paralysis is to be ascribed. The same state is further manifested by a violet or red coloration of the pulp and injection of vessels; varieties of colour resulting from inflammation, the only cause that can be imagined to maintain in the brain a continued vascular plethora. 'We conclude (adds M. Calmeil) that the want of cohesion in the grey substance is the result of inflammation.' To phlegmasia under different circumstances, and in a different modification, he likewise ascribes the hardening of the convolutions observed in some rare instances of the same disease, although he does not agree with MM. Bouchet, Casauvieilh, and Bouilleau,* in maintaining that this hardening is the first degree of inflammation.

This softening of the grey substance peculiar to general paralysis, in which it is separated into laminæ, and the external adheres to the pia mater, is, however, strongly distinguishable from ordinary ramollissement of the brain, in which its

---

* Bouilleau, Traité de l'Encephalite, et Archives Génerales de Médecine, t. viii.

substance is really diffluent, and its component particles have lost all their organic texture and relation. Whatever affinity there may be in the pathological causes of these two states, they are distinguishable in appearance and different in results.

The consistence of the white substance is generally normal or natural; in some few instances, as above hinted, it is harder than usual in the convolutions.

Besides the appearances resulting from high vascular injection, there is a discoloration of the grey substance which is a peculiar phenomenon. This appearance has been observed by M. Lallemand. It is attributed by M. Calmeil, as connected with general paralysis, to inflammation of the cortical substance, which he considers as the proximate cause of the disease.

7. This inflammation extends, as it would appear, to the ventricles, as the rugous or roughened state of the internal membrane, owing to subjacent villosities, indicates. These villosities and the membrane itself are often of a bright red colour. The appearance of inflammation is generally more marked in the fourth than in the lateral ventricles.

8. Local and particular lesions in the substance of the brain, such as apoplectic cysts, and likewise partial ramollissement, sometimes found in the medulla spinalis, are considered by M. Calmeil as merely accidental, and peculiar to the occasional disorders complicated, in particular instances, with general paralysis.

The conclusions resulting from these observations are very clearly deduced and expressed by the author. He says—

'The changes discovered in the heads of persons who have perished under general paralysis, viz. injection and absorption of the bony structure; injections of the dura mater; separation of its fibres; effusions of serosity into the cavity of the arachnoid; false membranes, organized or without organization; cysts filled with blood between its two laminæ; simple hemorrhages in the arachnoid; œdema of the meninges; injections and thickenings of the membranes; vegetations of the pia mater; development of their vessels; adhesions between the pia mater and the convolutions; disappearance of the grey substance; softening, hardening, and discoloration of the same substance; consistence and injection of the white substance; redness and tumefaction of the ventricular villosities; serosity in the ventricles, apoplectic cysts, erosions of the convolutions; ramollissement of the brain or of the spinal marrow;—these phenomena do not sufficiently explain the symptoms observed during life. The changes enumerated are so various in their appearance, and so far from uniform in occurrence, that they cannot on this account be immediately connected with the results.' M. Calmeil considers them all to be evidences of inflammation. This he infers to be the identical state of the brain, *of which the several appearances are diversified signs*, only varied manifestations of one and the same disease. 'Nearly all these disorders,' as he says, 'indicate a chronic phlegmasia in the brain, producing an identical modification, of which *the morbid appearances are only symptoms.*' "* 219.

In this last conclusion of M. Calmeil—namely, that the post-mortem changes are effects, and not causes, we entirely agree with M. Calmeil.

Our author then gives us the observations which Foville has made on the morbid changes in the brain and membranes, for the particulars of which we must refer to the volume itself. His two principal inferences, however, we may adduce.

" 1st. Morbid changes in the cortical substance are directly connected with intellectual derangement.

---

* Calmeil, p. 416.

2d, Morbid changes in the white substance are directly connected with disorders in the motive powers." 226.

In the second section of this chapter, Dr. P. examines into the state of the lungs in insanity. In 168 cases of melancholic derangement, M. Esquirol found only two instances of disease of the liver, while there were 65 of disease of the lungs. The same writer has computed that, in two cases out of eight of mental alienation, there will be found some disease of the thoracic viscera. In respect to the first computation, we may observe that, throwing insanity overboard, there will be found 65 cases of pulmonary disease for two cases of *disease* of the liver. So of the second computation—if one-fourth of the human species die of consumption, are we not likely to find two cases in eight of pulmonary disease after death, whether the patient die with the symptoms of insanity or not? This section is sheer nonsense—and the same, indeed, may be said of several other sections that follow. The probability is, that affections of the abdominal viscera, especially of the stomach, intestines, and liver, are far more frequently connected, in the light of causation, with insanity, than any other diseases of remote parts. But still *these* would not produce insanity, unless the brain or its membranes became involved—consequently, they could only be looked upon as predisposing the organ of the mind to disease. The following passage is the most sensible in these sections.

"The rare occurrence of organic diseases of the liver in cases of mental derangement is no proof, as M. Guislain has remarked, that pathologists have been always in error when they have conjectured or inferred from circumstances, that the functions of the same organ have been in a disordered state, and even that such disorder has had a considerable share in evolving the train of morbid phenomena which refer to the mind." 233.

The sixth chapter is on the theory or pathology of mental derangement; and the opinions of various writers are examined. In this, and, indeed, in all countries, the popular opinion is, that insanity is a malady of the mind itself, and that it may exist independently of any corporeal disorder. Such opinion is entertained even by some professional men on the Continent; but it is totally abandoned in England and France by all enlightened physicians. We have already hinted our own conviction, that insanity depends on corporeal disorder—the immediate seat of that disorder or disease being in the brain or its membranes. That the cerebral disorder may be produced by visceral disorders at a distance, we can have no doubt. The inflammation of a compound fracture will often produce delirium—and we know that dyspepsia will produce hypochondriasis—but still, in the one case and in the other, the immediate seat of the delirium and of the hypochondriasis is in the head. The nature of the cerebral affection, giving rise to insanity, is not so well ascertained as the seat of the complaint. Dr. P. seems inclined, on the whole, to consider it as allied to, if not actual inflammation. We are disposed to think, that *irritation* has often more to do in the pathology of insanity than *inflammation*. We do not deny, however, that the *latter* does not frequently co-exist with, or supervene on, the *former*. This brings us to the 7th chapter, on—

### Treatment of Insanity.

Our author follows the usual division, into moral and medicinal treatment of the insane, beginning with the medicinal, which consists of two indications, viz.—1st, to remove or lessen the diseased condition of the brain, on which insanity depends—and, 2dly, to restore and maintain a healthy condition of the physical or natural functions, and to obviate or remove disorders in other parts of the system connected with, or consequent on, diseased condition of the brain—in other words, chronic insanity, as contrasted with the acute stage.

*First Indication.* Our readers are prepared for the general tenor of treatment in the first stage of insanity, when inflammatory action, or vascular excitement, is taken as the proximate cause of the disease. The following qualifying cause is deserving of record.

" From the fact that the proximate cause of madness is nearly allied to inflammation, it does not follow, with certainty, that the disease is to be cured by the simple use of antiphlogistic remedies. The physician who would proceed to treat cases of madness as instances simply of inflammation in the brain, and who would expect to cure it at once, like any other local inflammatory disease, by the direct operation of antiphlogistic means, would very often find himself greatly disappointed. He would meet with many cases in which no perceptible benefit arises from bleedings, and evacuations of all kinds generally or locally applied, and combined with the whole series of remedies supposed to be required by the existence of organic inflammation. Many patients would sink under such a course of treatment, if carried on incautiously : it would leave the disease undiminished, and exhaust the powers of life. This depends, perhaps, on the influence of diseased states in other structures and organs, or on disordered functions of other parts which are complicated with, and in some instances give rise to, the disturbance existing in the brain. Inflammatory excitement is a part of the disease, but does not entirely constitute it." 252.

We are ready to admit, however, with Dr. Prichard, that there are few cases of insanity, where the practical indication arising from this view of the pathology will not be found applicable during some period of the malady —more especially in recent invasions of a violent nature, and where the patient is young and vigorous, with the usual symptoms of vascular excitement. The opinions of various authors are next adduced, as to the different items of the antiphlogistic treatment. Dr. Cullen advocated *bleeding*, to which Pinel was opposed, considering the signs of vascular plethora in the head as very deceptive, and, when acted on, as generally productive of mischief instead of benefit. Esquirol coincides with Pinel.

" He says that he has seen madness increased after an abundant flow of the catamenia, and likewise after one, two, or three bloodlettings. In such cases, melancholy dejection has passed into furious madness. Yet M. Esquirol approves of moderate bleeding in plethoric cases, and where some habitual sanguineous evacuation has been suppressed. He has often with advantage applied leeches behind the head or to the temples of patients who are subject to sudden determinations of blood towards the head. His favourite remedies in such cases were the use of a few leeches at a time, repeated as often as necessary, and cold applications to the head." 254.

Dr. Haslam advocates bleeding as the most beneficial remedy in insanity —being equally efficacious in melancholic as in maniacal cases. " He limits its use to recent cases and plethoric habits, and directs it to be performed by the application of six or eight cupping-glasses to the shaven scalp. From eight to sixteen ounces may be drawn, and the operation repeated as circumstances may require." Our readers will perceive that these limitations to "recent cases and plethoric subjects," reduce the measure of very mediocre pretensions. Dr. Rush, of Philadelphia, carried venesection to a great height; but his example will not be followed in this country. All his measures were of a most vigorous character; and, being applied to subjects of the new world, in a warm climate, and under moral and physical circumstances considerably different from those in the old hemisphere, they can hardly be adduced as models for imitation at present. From a patient, 68 years of age, he caused 200 ounces of blood to be drawn in less than two months. Foville steers a middle course.

"In the greatest number of cases of recent insanity which have been placed under my care, I have employed evacuations of blood, local or general, rare or frequent, abundant or in moderation, according to the strength of the patient, and the state of the pulse, the redness of the eyes, the heat of the head, the agitation and want of sleep." 257.

Dr. P. himself was led to adopt the practice of Foville, long before he heard of that gentleman's sentiments. He condemns, however, the inordinate detractions of blood recommended by Dr. Rush. The indications for phlebotomy in mental derangement, are those which point out an approach to phrenitis.

"I have stated my opinion on the subject of bleeding with the confidence which appears to myself to be the result of long and repeated experience of its beneficial effect; but I must not omit to remark that, although a majority of physicians who make insanity an object of study coincide with me, unless I am greatly mistaken, in this respect, there are others who hold an opposite opinion. Among these are some who have the very best claim to the confidence of the public, namely, that of more than ordinary success in the treatment of insanity. I am informed by Mr. Hitch, that at the Gloucester Lunatic Asylum, which is under the superintendence of Dr. Shute and his own immediate care, the use of the lancet, leeches, cupping-glasses, blisters, drastic purgatives, the practice of shaving the head, are totally proscribed. Yet among the patients admitted to this hospital, a very large proportion of recoveries, as I have already observed, take place, and *no cases* of sudden apoplexy or hemiplegia have yet happened." 261.

The above is worth alluding to.

The abstraction of heat from the head by evaporating lotions will generally be found useful in almost all cases of mania, especially where there is much excitement or violence. Affusion of cold water on the head has been recommended strongly by many experienced practitioners.

"A method of bathing adopted by M. Foville in the hospital under his management is free from the inconveniences and occasionally injurious results attendant on cold affusions. He places a cap or bonnet, containing ice and closely fitting, on the head of the patient, and keeps the body immersed in a warm-bath for two or three hours, and renews this proceeding twice or three times in a day, according to the intensity of symptoms. On adopting it, as he was accustomed to do at first, only once in a day, he found the tranquillity produced by it followed not unfrequently by increased agitation; but on repeating the bath, with the ice constantly applied to the head, he has frequently succeeded far beyond his expectation. 263.

On the remedial processes of counter-irritation, some useful, but common remarks are made, and then PURGATION is introduced, From the earliest records of medicine—witness the celebrity of Antecyra and Hellebore—purgation has held a high place in the treatment of insanity. Some caution is necessary in the administration of purgatives, since the mucous membrane of the intestinal canal is sometimes in a state of irritation or inflammation, during the existence of insanity. Emetics were once universally employed in insanity—and indeed in almost every disease. They are now comparatively in desuetude. Nauseating doses of antimony, however, are often of great service. Digitalis is still employed by some practitioners, especially where there is much arterial action. It is a remedy on which little dependence can be placed.

" Opium is far from being a remedy generally admissible in cases of insanity; yet there are instances in which it is decidedly useful. Its adoption requires care and discrimination. We are not possessed of any precise rules by which the effects which may arise from it can be with certainty predicted." 269.

Hyosciamus is safer; but a less effectual sedative, where sedatives are actually indicated. Camphor has been much vaunted by Hufeland and some other physicians, but is now generally considered as inefficient. Stramonium and belladonna are not entitled to much confidence. A mild alterative course of mercury, continued till the gums are affected, is an important measure, considering how often the disease is connected with disordered condition of the liver and other glandular organs.

*Rotatory Motion.* The suggestion of rotatory motion, with the view of reducing the force of the circulation by occasioning nausea and faintness, has been traced to the time of Cœlius Aurelianus. Darwin, in modern times, made the proposal—and Dr. Cox brought it into actual practice. In La Charité it is used at this day, as also in some parts of Germany. The effects of it are analogous to those of sea-sickness, and consequently are of a very sedative nature. Those who have given it a " fair trial," speak favourably of the remedy. The rotatory swing is useful in a moral point of view. Its effects are so disagreeable that patients dread it, and this dread may often act as a curb on their passions and impetuosities.

In respect to medicinal treatment, our author observes that it must be chiefly applicable to the acute stage and the early period of insanity. In subsequent periods, the marks of previous determination to the head will have generally subsided, giving way to an opposite condition—one of atony or relaxation, bordering on serous effusion, or approaching to various other changes implying the consequences rather than the causes of the malady. The treatment, then, of the early stage, being antiphlogistic, that of the later periods is " to restore and maintain a healthy condition of the physical or natural functions—and to obviate or remove disorders in other parts of the system."

" The relation in which these diseases stand to the cerebral disorder may be doubtful in many instances; in some it is the relation of cause, in others that of effect: even in the last instance there is a reaction of the secondary upon the primary parts in the series of morbid changes, and the original disease is aggravated by its complication with an accessory one." 275.

We need not advert to the various means of correcting various disorders of different organs. These will suggest themselves to every practitioner of any experience—and these are those who compose the bulk of our readers.

The Second Section of this Chapter is on "MORAL TREATMENT."

It is hardly necessary to remark, in limine, that the state of individuals, in the several forms and varieties of insanity, differs so much as to baffle all attempts at laying down fixed rules for their moral treatment.

"Maniacs, for example, who are under strong excitement, during paroxysms of raving madness, require personal coercion and even strict confinement of body and limbs in order to prevent mischief to themselves and others. Such treatment would be quite improper for other classes of lunatics, or even for the same individuals at different periods." 279.

Our author endeavours to arrange his observations on the "MORAL TREATMENT" of insanity under the following heads.

"1. Of the propriety of secluding or confining the insane, and separating them from society.

2. Of other means of abstracting them from the morbid impressions and associations which may have excited and may foster their mental disease.

3. Of the moral discipline and personal control under which they ought to be placed.

4. Of the treatment of their understandings in relation to their illusions or the morbid impressions on their minds." 280.

The first head—that of SECLUSION, is treated of in this section, only as regards the recovery of the patient, and not in a medico-legal point of view. From long attention to, and observation of the effects of seclusion—or rather of isolation, in mental derangement, we are quite convinced that the cases where the said isolation is improper, form only a very few exceptions to the general rule. If a father, brother, or son were affected with this mental malady, we should not hesitate one moment in placing him in a proper lunatic asylum—and that not to diminish our risk or trouble, but for the sake of promoting the recovery of the patient.

False lenity in such cases is real cruelty. But the arguments are so well put in the following extract, that we shall place it in our pages, as a ready reference to shew to the friends of patients, when they are in doubt what course to pursue.

"M. Esquirol has observed that all English, French, and German physicians, who have devoted themselves to the study of mental diseases, recommend the confinement of the insane, and are unanimous as to the utility of this proceeding as means of cure.

'Cullen has pointed out the necessity of confining lunatics, and separating them from their relations and friends. Willis, who acquired so great celebrity by having assisted towards the happy termination of the first attack of madness experienced by George III., unfurnished the king's apartments, dismissed his courtiers and domestics, and had him attended by strange servants. Willis asserts that insane persons from the continent, who came to seek his advice, got well more frequently than his countrymen.

'M. Pinel, in his Treatise on Madness, his best title to the admiration and gratitude of mankind, has pronounced seclusion to be the foundation of all rational treatment of mental diseases.'

We here find this statement made in very general terms ; whether any and what limitations are required or were intended will be apparent, when we shall have considered the reasons which have been assigned for having recourse to such a proceeding.

The reasons which indicate the necessity of secluding and confining insane persons are briefly stated by M. Esquirol in the following heads.

1. The insane ought to be confined for their own security, for that of their families, and for the maintenance of public order. This consideration is not within the scope of our present inquiry.

2. To remove them from the influence of external circumstances which may have produced their disorder, and may be likely to protract it.

3. To overcome their resistence to curative means.

4. To subject them to a regimen appropriate to their situation. And

5. To cause them to resume their moral and intellectual habits.

That the second of these objects, which is the foundation of all curative proceedings, can only be obtained by withdrawing insane patients from their homes, and secluding them from their families and society, becomes apparent on considering the prevalent state of the feelings and affections of lunatics, and the results arising from the erroneous impressions which cloud their understandings.

'The sensibility of the insane,' says M. Esquirol, 'is perverted: they no longer have any relations with the external world but those of a disordered and consequently painful nature. Every thing irritates them, distracts them, and excites their aversion. In constant opposition to all that surrounds them, they soon persuade themselves that persons are combined to injure them; and neither understanding what is said, nor being able to comprehend the reasonings that are addressed to them, they misinterpret the most affectionate expressions and the wisest counsels; they mistake the most candid, serious, and tender language for insults, irony, and provocations, and the most attentive kindness for contradictions. The regimen and the prohibitions which are called for by their situation, and to which their attendants wish to subject them, appear to them cruel persecutions.

'The heart of the insane cherishes no feeling but mistrust; he is irritated to anger by every thing he sees, and is so timid and fearful that he is troubled as soon as any one approaches him. Hence arises the conviction that every one tries to vex, defame, ruin, and destroy him. This conviction puts the finishing stroke to the moral perversion of his mind, and from it arises that symptomatic mistrust, which is observed in all the insane, even in maniacs who appear so bold and audacious.

'From mistrust these patients soon pass to fear or hatred; and in these new moral situations they repel their relations and friends, and welcome strangers, throwing themselves into their arms, calling them their protectors or liberators, with whom they are ready to fly, and abandon their home and family.

'With these moral dispositions, if left in the bosom of his family, the tender son, whose happiness used to consist in living near his mother, and in following his father's counsels, persuaded that they wish to disgust him with his home in order to drive him from it, falls into the deepest despair, or escapes to destroy himself.' 'Another unhappy person becomes, all at once, lord of the world, dictates his sovereign commands to all that surround him; he expects to be blindly obeyed by all those who have been accustomed to yield to his will through respect or affection. His wife, his children, his friends, his servants, are his subjects; they have hitherto always submitted to his will: how dare they resist him now? He is in his own territory; his commands are despotic; he is ready to punish with the greatest severity whoever shall make the least remonstrance. What he requires may be impossible—that is of no consequence: should the commands of the all-powerful meet with obstacles? The affliction of his family, the chagrin of his friends, the anxiety of all, their deference to his will and caprices, and the repugnance that each evinces to oppose him from the fear of exasperating his fury, contribute to confirm him in his imaginary possession of power and dominion. Withdraw him from his pretensions, transport him far from his house, from his empire,—removed from his subjects, surrounded by new scenes, he will collect his ideas, direct his attention to himself, and place himself on an equality with his companions." 283.

The preceding observations will be found applicable perhaps to all maniacs—and even to many of those who are monomaniacal. In mania, or raving madness, strict confinement is obviously required. And in most cases of monomania the understanding is so disturbed, and the moral affections so perverted, that no alternative is left but isolation. When the disorder of the understanding is so restricted as to leave to the patient the exercise of a great portion of his reason, the case is more difficult of adjudication. It seems cruel to deprive him of the attention of his family, and separate a miserable being from the objects of his affections. M. Esquirol candidly admits that isolation is not invariably useful in these cases.

"When the predominant feelings of the monomaniac are such as are calculated to estrange him from and to set him at enmity with his relatives; when he is actuated by pride and misanthropy, by jealousy, hatred, malice, confinement is absolutely necessary. If, on the contrary, the illusion of the insane relates to objects of indifference, and excites no strong emotions; if he has no aversion to his home and the persons with whom he lives, although confinement may be sometimes useful, it is not absolutely necessary. 'But if the patient, retaining a large portion of his intellect, has a strong attachment to his relatives, it is to be feared that confinement might aggravate the disease." 288.

The risk of suicide which is constantly run in cases even of monomania, is a great inducement to place the individual in security. Many a horrifying scene of this kind would be prevented by timely isolation. A troublesome description of patients are those where mental derangement follows inebriation. When placed in an asylum and deprived of drink, they soon recover. They demand their liberty—return to their old habits—and an aggravated attack of insanity is usually the consequence. Confinement, in such cases, would be the surest prophylactic, but, in this country, it could not be put in force.

The period at which the insane should be liberated is often very difficultly determined. Great care is necessary not to dismiss the insane too soon, as they often exhibit considerable art in disguising their real condition. Even when recovery is complete, a strong predisposition to relapse exists—which relapse is readily induced by any sudden excitement, intemperance, or gust of passion.

"M. Pinel has given some important remarks on this subject, which I shall briefly abstract. He observes that any sudden alarm, transport of anger, or of grief; that intemperance, hot weather, or even a sudden change from a state of confinement and constraint to liberty, is liable to produce in convalescent lunatics a disturbance of which they would not be susceptible in other circumstances, and to renew the attacks of mania when the habit has not been long suspended: that some convalescent patients, who have been taken away too soon by their friends have suffered relapses, and have been obliged to return several times to the hospitals. A grenadier of the French guards, who was one of the foremost to mount the Bastile at its assault, gave himself up to the most unbounded ambition, was disappointed in his brilliant expectations, and fell into the most violent maniacal delirium. He remained four months in this state of fury and confusion after his arrival at Bicêtre. When he became calm, his mother took him away before his reason was confirmed: hence he experienced a return of his attacks in the midst of his family, and it became necessary to remove him again to the hospital. The same imprudence was renewed twice with the same results. His mother, then instructed by experience, was no longer anxious to remove him unseasonably: he passed two years tranquilly and without any attack, left the hospital at the beginning of the winter, and never again experienced a return of his disease." 291.

This part of the work concludes with some general observations on exercise, and on some other points of moral management not included in the preceding chapters. Here we must terminate our analysis. There are three or four more chapters in the work, occupying about 180 pages, on puerperal mania, idiotism, statistics of insanity, medical jurisprudence as it relates to insanity, gestatic affections—and supplementary notes on phrenology, the forms of skulls, and the functions of the brain. Some of these subjects we may take up in another article, as they are not necessarily connected with the present subject, as far as an analytical review of the book is concerned.

The copious account which we have here presented of Dr. Prichard's work renders any summary opinion of it unnecessary. It is a laborious and judicious compilation, containing, perhaps, as much original matter as was necessary or beneficial in a production of this kind. The author is entitled to great respect for his opinions, not only because he is well known as a man of extensive erudition, but also on account of his practical acquaintance with the subject on which he writes. The work, we may safely say, is the best, as well as the latest, on mental derangement in the English language.

